# Prevention and treatment strategies for contextual overgeneralization

**DOI:** 10.1038/s41598-017-16893-2

**Published:** 2017-12-05

**Authors:** Dieuwke Sevenster, Kim Haesen, Bram Vervliet, Merel Kindt, Rudi D’Hooge

**Affiliations:** 1 Laboratory of Biological Psychology, Department of Psychology, KU Leuven, Tiensestraat 102, B-3000 Leuven, Belgium; 2 Center for Excellence on Generalization, Department of Psychology, KU Leuven, Tiensestraat 102, B-3000 Leuven, Belgium; 3 Clinical Psychology, Utrecht University, Heidelberglaan 1, 3584 CS Utrecht, The Netherlands; 4 Center for Learning Psychology and Experimental Psychopathology, Department of Psychology, KU Leuven, Tiensestraat 102, B-3000 Leuven, Belgium; 5 Department of Psychiatry, Harvard Medical School at Massachusetts General Hospital, 149 13th St, Charlestown, MA 02129 USA; 6 Department of Clinical Psychology, University of Amsterdam, Nieuwe Achtergracht 129, 1001 NK Amsterdam, The Netherlands; 7 Amsterdam Brain and Cognition, Nieuwe Achtergracht 129, 1001 NK Amsterdam, The Netherlands

## Abstract

At the core of anxiety disorders lies the tendency to generalize fear from a threatening to a safe situation. A deeper understanding of the mechanisms that facilitate and restrain generalization in humans is therefore needed. Rodent studies showed that pre-exposure to a context that is similar to the threatening context enhanced generalization to the similar context. In Experiment 1 we replicated these animal findings in humans (US-expectancy). Studies on the underlying mechanisms showed that the pre-exposure representation was recalled during conditioning (due to similarity between the contexts) and the shock also became linked to the recalled representation, resulting in greater generalization. In Experiment 2 we developed a pre-exposure procedure that increased the ability to distinguish between the conditioned and pre-exposure contexts, such that presentation of the former would no longer result in recall of the latter. We then observed that overgeneralization (US-expectancy) was prevented. Pre-exposure did not affect generalization of skin conductance response or fear potentiated startle. Finally, exploratory analyses revealed that increased generalization (US-expectancy), if not prevented, could be reduced by a reminder of the conditioned context. Hence, we developed a prevention- and a treatment-strategy for overgeneralization. These findings may guide the development of new therapeutic strategies.

## Introduction

Generalization of fear is a defining feature in the onset and maintenance of anxiety disorders^1–3^. Following a traumatic event (e.g., being robbed in a dark ally), an individual comes to regard a situation that is actually safe but related to the threatening experience (e.g., all dark streets) as potentially harmful. Unrestricted fear generalization can seriously impair social functioning and quality of life (e.g., never leave the house after dark). A better understanding of the mechanisms underlying fear generalization could stimulate the development of new strategies to curb generalization.

Basic neuroscience studies demonstrated that neutral or non-threatening experiences before fear learning could heavily influence subsequent fear generalization; one day before conditioning in context A, rodents were pre-exposed to a context that was similar (context B) to the threatening context (context A), resulting in increased generalization to that previously neutral context^4–6^. It is important to note that pre-exposure did not necessarily result in overgeneralization; it did not occur when animals were pre-exposed to a context that was completely different from the conditioned context, suggesting that similarity between contexts is crucial^4–6^. So far it has not been demonstrated that overgeneralization through context pre-exposure can also be observed in humans. In the first experiment we will investigate whether pre-exposure to a similar context enhances generalization at test.

The phenomenon of pattern completion is crucial to understand pre-exposure-induced overgeneralization. Pattern completion refers to the ability to recall a memory representation with a partial cue^7,8^. In general, presentation of a context that shares features with a previously presented context results in recall of that context. More specifically, exposure to the conditioned context triggered recall of the similar pre-exposure context, due to the overlap in features between contexts. The representation of the pre-exposure context was thus active during conditioning, resulting in a link between the recalled pre-exposure context and shock. This link between the pre-exposure context and the shock resulted in increased generalization to the similar contexts at test. Crucially, prevention of recall of the neutral pre-exposure context during conditioning would eliminate overgeneralization all together. Here, we aimed to demonstrate that pre-exposure- induced overgeneralization could be prevented by manipulating the pre-exposure session. (Note that studies have been conducted that also made use of pre-exposure techniques to reduced generalization^9^. These studies aimed to reduce over generalization as a result of the passage of time. But here we specifically aimed to design a technique that prevents the overgeneralization induced by pre-exposure to a similar context).

Perceptual learning research may offer such a preventive strategy. There is an abundance of evidence showing that pre-exposure to a pair of similar stimuli increases the ability to discriminate between those stimuli^10–12^. Subjects were exposed to the common features twice as often as to the unique elements of the stimuli. The common elements became less salient while the unique elements became more distinct. In agreement, pre-exposure to two similar contexts will result in the increased ability to discriminate between the unique features of similar contexts. Then, presentation of the conditioned context will result in discrimination rather than recall of the similar context. We investigated whether pre-exposure to two similar contexts could be used as a preventive strategy for fear generalization. Note that we chose to pre-expose participants to two contexts that were similar to the conditioned context but not the conditioned context itself. We would expect that pre-exposure to the conditioned context and the similar context also prevents overgeneralization. However, in real life it is difficult to foresee the exact threatening context (i.e., before being send on a military mission). Hence, it would be more feasible to pre-expose an individual to contexts that resemble the context in which a traumatic event might take place.

In sum, the first aim of the study was to replicate animal findings that pre-exposure to a similar context could result in increased generalization in humans (Experiment 1). The second aim of the study was to investigate whether this overgeneralization could be restrained. Building upon insights from animal experimental research we investigated a preventive strategy for overgeneralization that is both neuroscience-based and non-invasive (Experiment 2). Finally, outside the lab, prevention is not always feasible (e.g., after trauma has taken place). Therefore, after pre-exposure has induced overgeneralization, strategies to reduce generalization are required. This could be achieved by stimulating the ability to discriminate between the conditioned context and the similar context at test. Insights from studies aiming to reduce overgeneralization as a result of the passage of time will be used to this end. Animals were conditioned in context A and tested for generalization in context B after several weeks. Typically, a loss of contextual features and overgeneralization to context B is observed at that time^13,14^. A return to the conditioned context A before test in B reduced generalization to that context. This reminder of the conditioned context recovered memory accuracy and increased the ability to discriminate between the conditioned context and the similar generalization context^13^. Similarly, we subjected the data collected in Experiment 2 to exploratory analyses to investigate whether a reminder of the conditioned context before generalization test could reduce pre-exposure-induced overgeneralization.

## Experiment 1. Pre-exposure induces contextual overgeneralization in humans

Animal studies robustly demonstrated that pre-exposure to a context that is similar (but not the same or different) to the conditioned context enhanced generalization to that context^4–6^. To replicate these findings in humans, we employed a 3-day context-conditioning paradigm. On day 1, participants were pre-exposed to one of three contexts: the to-be conditioned context (context A), the similar context (context B), or the different context (context X). The next day (day 2) context conditioning (context A) took place. Finally, test in the conditioned context (context A),the similar context (context B), and the different context (context X) took place on day 3. We hypothesized that pre-exposure to a similar context (context B) would result in overgeneralization. This was compared to the effect of pre-exposure to the same context (context A) or a completely different context (context X). We expected that generalization from the conditioned context (context A) to the similar context (context B) would be enhanced when pre-exposed to the similar context. Overgeneralization would be evidenced by an absence of a difference between responding in the conditioned context and the similar context on test. In contrast, we expected discrimination between the conditioned context and the similar context when pre-exposed to the same (context B) or a different context (X).

To rule out that pre-exposure per se is not sufficient to enhance generalization, participants were also tested in context X. We did not expect that pre-exposure to the different context (context X) would enhance generalization to context X on test, given that similarity between the pre-exposure and conditioned context is crucial for overgeneralization^4–6^. Finally, we hypothesized that overgeneralization as a result of pre-exposure to a similar context B is restricted to that context. Previous animal studies showed that the pre-exposure representation becomes specifically linked with shock during conditioning and therefore generalization to a different context was not observed^4^. Hence, we expect that pre-exposure to a similar context would not result in overgeneralization to the different context X.

## Methods and Materials

### Participants

Forty-two (24 female; 18 male) healthy undergraduate students participated in the study, ranging in age between 18 and 25 years (*M* = 21.24, *SD* = 1.50). Participants received either partial course credit or a small amount of money (32 euros) for their participation. All participants gave informed consent and were notified that they could withdraw from participation at any time. The experimenter briefly screened the participants to assure they were free from a physical (i.e., heart disease or epilepsy) or psychiatric disorder. The study was approved by and in accordance with the guidelines of the Ethics Committee of the University of Amsterdam. Participants were randomly assigned to the pre-exposure A (*n* = 14), pre-exposure B (*n* = 14), or the pre-exposure X (*n* = 13) condition. Of these participants, two (pre-exposure X condition) were excluded from analysis of day 2 FPS responding due to technical difficulties. One participant (pre-exposure A condition) was excluded from all analysis due to technical difficulties.

### Apparatus

#### Stimuli

**Figure 1:**
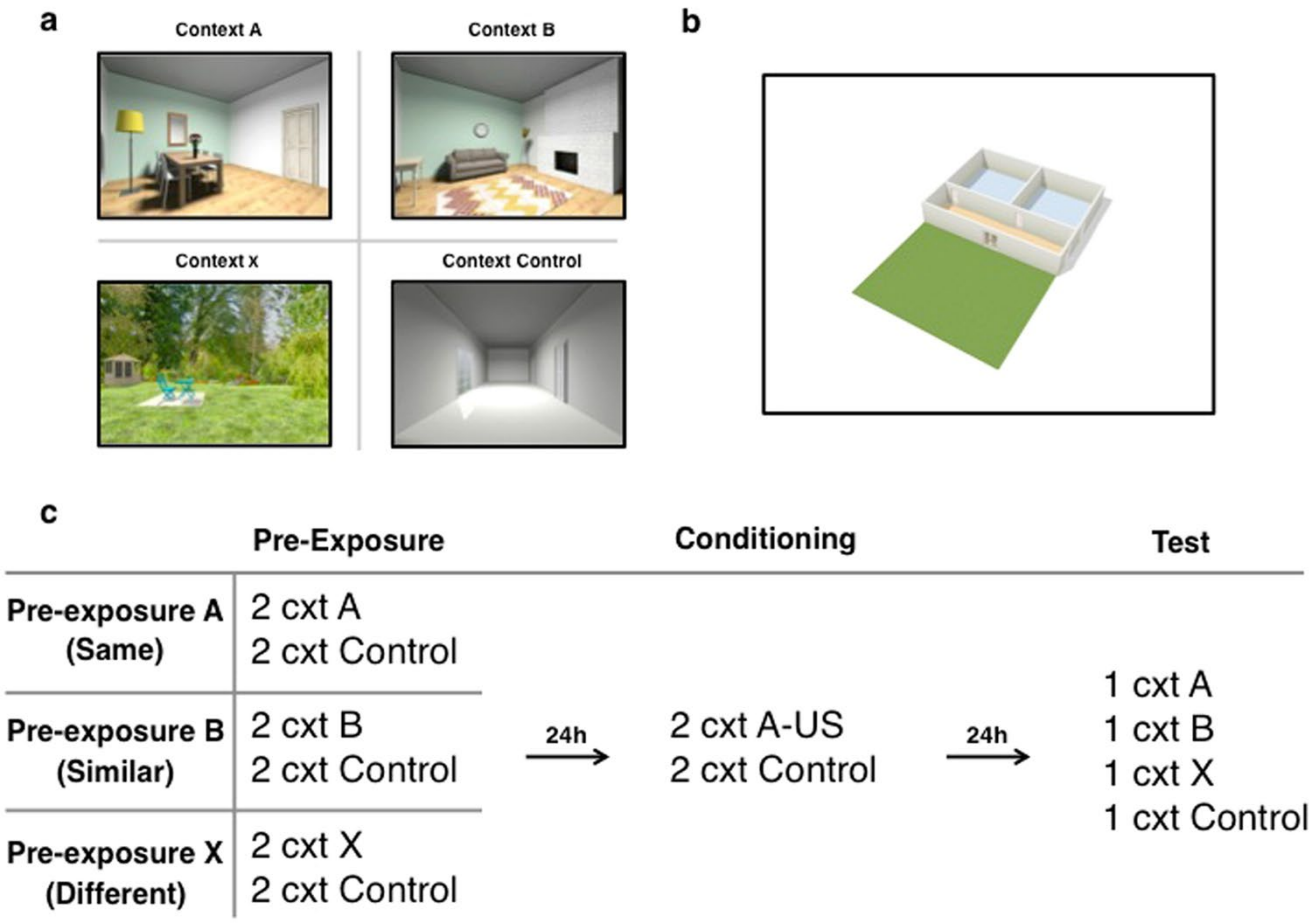
Overview of stimuli and experimental procedure for Experiment 1. Contextual stimuli (**a**), floor plan of the house (**b**), and schematic representation of the experimental design (**c**).

Participants were presented different areas of a house. A floor plan of the house was presented before and throughout the experiment. The contextual stimuli were static images of different rooms and a garden of a house (Fig. 1a,b). Two living room images served as context A and B, whereas an image of the garden served as context X. The living room images (context A and B) shared common features: the floor and the wall were the same for context A and B, as was the point of view of the living room images. Differences between the rooms consisted of furniture and different locations on the floor plan. There was no overlap in features between the living rooms and the garden (context X), which was located next to the house on the floor plan. Context A served as the threatening context (i.e., conditioned context), context B as the similar context and context X as the context completely different from the threatening context. An additional context served as context control, consisting of an image of a hallway. Assignment of the living room images as context A or B was counterbalanced across all participants. A floor plan of the house was shown at the beginning of the experiment. The contextual stimuli and the floor plan were created by interior designing programs (www.roomstyler.com; www.floorplanner.com). Stimulus presentation was controlled by Presentation software (Neurobehavioral Systems, www.neurobs.com).

#### Unconditioned stimulus

The US was a 2ms electrocutaneous stimulus administered to the wrist of the left hand. Electrical stimulation was delivered through a pair of Ag electrodes of 20 by 25 mm with a fixed inter-electrode mid-distance of 45 mm. Shock were delivered by a Digitimer DS7A constant current stimulator (Hertfordshire, UK). Between the electrodes and the skin a conductive gel (Signa, Parker) was applied^15–18^.

#### Fear potentiated startle (FPS)

Startle response was measured through electromyography (EMG) of the right orbicularis oculi muscle. Two 6 mm sintered Ag/AgCl electrodes filled with a conductive gel (Signa, Parker) were positioned approximately 1 cm under the pupil and 1 cm below the lateral canthus, respectively; a ground electrode was placed on the forehead, 1 cm below hairline^19^. The startle probe was a 40ms duration noise burst (104 dB) with a rise/fall time shorter than 1ms, which was presented binaurally through headphones (Sennheiser, model HD 25-1 II). The EMG signal was amplified in two stages. The input stage had an input resistance of 10 MOhm, a frequency response of DC-1500 Hz and an amplification factor of 200. The bandwidth of the EMG channels was set to 1500 Hz to maintain optimal time accuracy in the onset and offset of the EMG response. Because the signal power in the frequency range from 400–1000 Hz is very low^20^, aliasing effects due to the low sample frequency are negligible. An online 50 Hz notch filter was used to reduce interference of the mains noise. The second stage amplified the signal with a variable amplification factor of 0–100 x. The raw EMG signal was sampled at 1000 Hz. The data was band-pass filtered (28–500 Hz, Butterworth, 4^th^ order^19^) offline. The high-pass filter (28 Hz/4th order) was applied to remove movement artefacts and baseline fluctuation caused by electrode offsets, resulting in a very low resting state EMG level that remained constant during the experiment. Due to this low baseline level baseline correction was unnecessary and instead absolute values were used to reduce the negative effect of for example short eye blinks occurring just before stimulus onset. After rectifying and contour following (time constant = 10 ms) the peak amplitude was found by analysing the first derivative of the resulting signal in a 30–150 ms interval following probe onset.

#### Online US-expectancy ratings

Participants rated their US-expectancy online on an 11-point scale ranging from ‘certainly no electric stimulus’ (−5) through ‘uncertain’ (0) to ‘certainly an electric stimulus’ (5). The scale was placed at the bottom of the screen. US-expectancy was rated continuously throughout the 2 min of context presentation. Ratings were recorded at a rate of 2 samples per second. US-expectancy levels were rated by shifting the cursor on the scale with use of the mouse throughout the context presentation (no button presses). Subjects were not informed about contingencies and were instructed to update their US-expectancy throughout context presentation by moving the cursor or leaving it in place, as long as the position on the scale resembled their current expectancy.

#### Subjective assessments

State and trait anxiety was measured with the Trait Anxiety Inventory (STAI-T)^21^. Evaluation of the US was assessed on an 11-point scale ranging from ‘unpleasant’ (−5) to ‘pleasant’ (5).

### Procedure

The experiment consisted of a pre-exposure, conditioning, and test session on three consecutive days. For a schematic representation of the experimental design see Fig. 1c. Every test session started with ten startle noise alone (NA) trials to stabilize baseline startle reactivity. On the first testing day participants were screened and filled out informed consent and the trait anxiety (STAI-T) questionnaire. Next, they were seated in front of a computer screen in a sound-attenuated room. The EMG electrodes were attached. To create a neutral experience for the pre-exposure session, the US-electrodes were not attached. Consequently, US-expectancy was not rated on the first day of testing. Before actual presentation of the contexts participants were shown the floor plan of the house and were instructed that they would visit different areas of the house. While the images were static, we aimed to create an experience of being led through the different areas of the house. To this end, the floor plan was used to underline that the areas belonged to one house. Before presentation of a context image, the area the participant was about to see was highlighted on the floor plan and participants were instructed that they were about to enter a room/hallway/garden of the house. Before every context presentation the floor plan was shown again and the context they were about to see was highlighted. During pre-exposure participants were first presented the control context, followed by the pre-exposure context (block 1). This was repeated once (block 2), making a total of two presentations of the control context and two presentations of the pre-exposure context. Contextual stimuli were presented for 2 minutes. Ten seconds after context onset the first of three startle probes (40ms; 104 dB) during context presentation was delivered, with an inter-probe interval of 40 s. Participants in the pre-exposure A group were pre-exposed to the to-be threatening context; participants in the pre-exposure B group were pre-exposed to the context that was similar to the threatening context; participants in the pre-exposure X group were pre-exposed to the context that was completely different from the threatening context.

The next day the procedure was the same for all participants. The EMG and shock electrodes were attached and US-intensity level was determined by gradually increasing shock intensity (starting at 1 mA) until participants indicated the shock to be ‘uncomfortable though not painful’. Participants did not receive information about the relationship between the contexts and the US. Expectancy was rated throughout presentation of the context. They were instructed to continuously place the cursor of the mouse on the position on the scale corresponding to their US-expectancy, ranging from ‘certainly no electric stimulus’ (−5) through ‘uncertain’ (0) to ‘certainly an electric stimulus’ (5). First the control context was presented followed by the threatening context (block 1), which was repeated once (block 2). During the first presentation of the threatening context 4 USs were delivered at 16s, 38s, 63s, and 105s after context onset. To make US delivery as unpredictable as possible three USs were presented at different time points (28s, 70s, 80s after context onset) during the second threatening context presentation. After conclusion of the experimental phase participants rated US-pleasantness. As on day 1, startle probes were presented and before presentation every context was highlighted on the floor plan.

One day later participants returned for the final testing day (day 3). The EMG and shock electrodes were attached and participants were instructed that the experiment would be continued. Every context was presented once. The control context was presented first, followed by random presentation of contexts A, B, and X. Again, participants rated their US-expectancy, startle probes were presented and every context was highlighted on the floor plan before presentation. On day 3 no shocks were delivered.

It is important to note that in contrast to the animal studies^4,5^, but in line with human context conditioning studies^22,23^, we employed a differential conditioning paradigm. This allows the demonstration of learning by comparing responses to the threatening context to responses in the control context. The control context was, thus, added to control for responding in the conditioned context (day 2 and day 3). Note that generalization was assessed by comparing responding in the conditioned context and the generalization contexts on day 3.

### Data analysis

Startle responses were averaged for each context presentation. To control for inter-individual differences in startle responsiveness startle data were T-transformed. Day 2 (acquisition) US-expectancy ratings were averaged over the 2 min of context presentation. Day 3 (generalization test) US-expectancy ratings were averaged over the first 30 s of context presentation. FPS during pre-exposure on day 1 was tested with a mixed analysis of variance for repeated measures (ANOVA) with context (pre-exposure context; control context) and trial (trial 1; trial 2) as within-subjects factors; group (pre-exposure A; pre-exposure B; pre-exposure X) was included as between-subjects factor to demonstrate that the groups did not differ in startle responding during pre-exposure. US-expectancy was not measured during pre-exposure. To test acquisition of US-expectancy and FPS on day 2, data were subjected to a mixed ANOVA with context (context A; control context) and trial (trial 1; trial 2) as within-subjects factors; group (pre-exposure A; pre-exposure B; pre-exposure X) was included as between-subjects factor to demonstrate that the groups did not differ in acquisition. To demonstrate that differential US-expectancy ratings between the threatening context A and the control context persisted from conditioning (day 2) to test (day 3), and did not differ between groups, a mixed ANOVA with context (context A; control context) and trial (last acquisition trial day 2; test trial day 3) as within-subjects factors and group (pre-exposure A; pre-exposure B; pre-exposure X) as between-subjects factor was performed.

Further analyses were performed for both US-expectancy and FPS responding. To test for differences between groups in generalization from the conditioned context A to the similar context B on day 3 a mixed ANOVA with group (pre-exposure A; pre-exposure B; pre-exposure X) as between-subjects factor and context (context A; context B) as within-subjects factor was performed. Following up on a significant interaction with group, paired-samples t-tests (context A vs. context B) were performed for the groups separately. To test for generalization from the conditioned context A to the different context X on day 3, the same analyses were performed replacing context B with context X. The alpha level was set at 0.05 for statistical analyses. A Greenhouse-Geisser procedure was used in case of violation of the sphericity assumption in ANOVAs.

### Data availability

The programmed experiment (including stimulus materials) and the datasets analysed during the current study are available in the Open Science Framework (OSF) repository, https://osf.io/2dcp4/?view_only=05f27542f4144497893f2c94453b3857.

## Results

### Participants

There were no differences in US-intensity, US-evaluation and reported state and trait anxiety (*F*_s_ < 1) between the groups (Table 1).

**Table 1:** Mean values (SD) of the US-intensity, US-evaluation, state anxiety (STAI-S), and trait anxiety (STAI-T) for the pre-exposure A (*n* = 14), pre-exposure B (*n* = 14), and pre-exposure X (*n* = 13) groups.

	Pre-exposure A	Pre-exposure B	Pre-exposure X
US-intensity (mA)	23.4 (10.9)	19.2 (12.5)	19.0 (11.2)
US-evaluation	−2.6 (2.1)	−2.78 (0.8)	−2.5 (1.3)
State anxiety	32.7 (6.2)	31.8 (7.1)	30.5 (7.1)
Trait anxiety	35.6 (7.4)	37.9 (10.8)	34.1 (6.3)

### US-expectancy ratings

For analyses on differential responding between the threatening context and the control context during conditioning (day 2) and test (day 3) see Supplementary materials.

#### Generalization to the similar context (context B)

**Figure 2:**
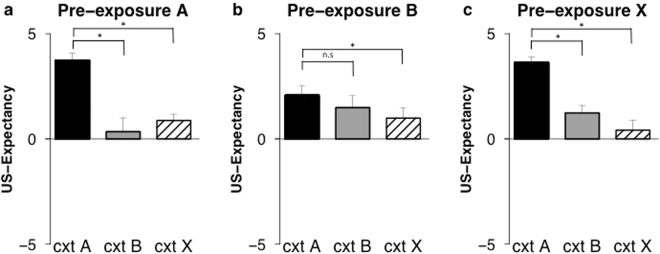
Pre-exposure to a similar context enhances generalization of US-expectancy. Mean US-expectancy ratings to context A, context B, and context X on test (day 3) for the pre-exposure A (*n* = 14) (**a**), pre-exposure B (*n* = 14) (**b**), and pre-exposure X (*n* = 13) (**c**) conditions. Error bars represent s.e.m.

Analyses revealed differences between groups in differential ratings to conditioned context A and the similar context B (context x group; *F*_(2, 38)_ = 7.97, *p* < 0.001, *η*²_p_ = 0.30) (Fig. 2a–c). Follow-up analyses showed overgeneralization when pre-exposed to the similar context B, evidenced by an absence of differences in ratings to context A and B in the pre-exposure B group (*t*_(13)_ = 1.19, *p* = 0.26, *d* = 0.33) (Fig. 2b). In contrast, there were higher US-expectancy ratings in the conditioned context compared to the similar context B in both pre-exposure A group (*t*_(13)_ = 6.12, *p* < 0.001, *d* = 1.70) (Fig. 2a) and pre-exposure X group (*t*_(12)_ = 5.14, *p* < 0.001, *d* = 1.77) (Fig. 2c). Hence, in line with our expectations we observed overgeneralization from the conditioned context to the similar context in those participants that were pre-exposed to the similar context B (pre-exposure B condition). Those participants pre-exposed to a context that was the same (pre-exposure A condition) or completely different (pre-exposure X condition) from the conditioned context showed good discrimination between the conditioned context and the similar context at test.

#### Generalization to the different context (context X)

Contrary to expectation, analyses showed differences between groups in generalization of US-expectancy ratings from conditioned context A to the different context X (context x group; *F*_(2, 38)_ = 5.63, *p* < 0.007, *η*²_p_ = 0.23) (Fig. 2a–c). To further investigate this unexpected effect, follow-up ANOVA’s were performed, comparing the groups to one another. There was reduced discrimination between the contexts (A and X) when comparing the pre-exposure A and pre-exposure B groups (context x group; *F*_(1, 26)_ = 7.37, *p* < 0.012, *η*²_p_ = 0.22). Similarly, there was reduced discrimination between the contexts when comparing the pre-exposure B and pre-exposure X groups (context x group; *F*_(1, 25)_ = 10.19, *p* < 0.004, *η*²_p_ = 0.29). The pre-exposure A and pre-exposure X groups did not differ (context x group; *F*_(1, 25)_ < 1, *p* = 0.63, *η*²_p_ = 0.01). Participants in the pre-exposure A group (*t*_(13)_ = 5.70, *p* < 0.001, *d* = 1.58) (Fig. 2a), in the pre-exposure B group (*t*_(13)_ = 2.57, *p* = 0.023, *d* = 0.71) (Fig. 2b), and in the pre-exposure X group (*t*_(12)_ = 6.15, *p* < 0.001, *d* = 1.77) (Fig. 2c) showed higher ratings to the conditioned context compared to the different context X.

Hence, US-expectancy was elevated in the conditioned context relative to the different context on test across all conditions. However, contrary to previous findings^4^, we did observe overgeneralization when pre-exposed to the similar context.

### Fear potentiated startle (FPS)

Unexpected differences in startle responding during pre-exposure made interpretation of the day 2 and day 3 startle data difficult (see Supplementary materials).

### Conclusion Experiment 1

In line with expectation, pre-exposure to the similar context (context B) facilitated generalization of US-expectancy to that context. Those pre-exposed to the threatening context (A) or a completely different context (X) showed good discrimination of US-expectancy between the conditioning context and the similar context on test. It is important to note that pre-exposure itself did not facilitate generalization, as US-expectancy in context X was not enhanced when pre-exposed to context X; as shown by previous studies, an overlap between contexts is a prerequisite for increased generalization. Contrary to expectation, we did observe overgeneralization of US-expectancy to the different context (context X) when pre-exposed to the similar context B. Thus, pre-exposure to the similar context not only resulted in overgeneralization to that context but also to a different context.

In sum, in the first experiment we demonstrated that pre-exposure to a similar context before conditioning enhanced generalization of US-expectancy to a similar and a different context. In the second experiment we will investigate how to restrain overgeneralization.

### Experiment 2. Preventing and reducing fear generalization in humans

In experiment 1 pre-exposure to a similar context promoted generalization to other contexts. Animal studies demonstrated that during conditioning the representation of the similar context is also retrieved, resulting in overgeneralization to that context^4–6^. Hence, if we would be able to prevent retrieval of the pre-exposure representation during conditioning, the increase in generalization would not occur. We aimed to stimulate the ability to discriminate between similar contexts during pre-exposure. Then, instead of recall of the pre-exposure representation during conditioning, discrimination between the pre-exposure context and the conditioned context would occur, thereby preventing overgeneralization.

In the second experiment, participants were pre-exposed either to two similar contexts (contexts B and C) or to the similar and the different context (contexts B and X). We hypothesized that pre-exposure to two similar context (contexts B and C) would prevent overgeneralization (Experiment 2). We expected discrimination between the conditioned context A and the generalization contexts B and X when pre-exposed to two similar contexts (context B and C). Furthermore, we hypothesized that pre-exposure to one similar and one different context (contexts B and X) would not prevent the increase in generalization.

Hence, in the group in which participants were pre-exposed to a similar and a different context we expected to still observe overgeneralization. We therefore also investigated whether, if not prevented, overgeneralization could be reduced. We hypothesized that a reminder of the conditioned context would enhance discrimination between the conditioned context and the generalization contexts. To this end, test order on day 3 was manipulated, such that half of the participants were first tested in the conditioned context A (Reminder), and half of the participants were first tested in the similar context B (No reminder). Post-hoc analyses were performed to investigate whether test order affects generalization. When pre-exposed to a similar and different context (pre-exposure BX group), we expected higher ratings in the conditioned context compared to the generalization contexts when participants were first tested in the conditioned context (Reminder); we expected to still observe overgeneralization when first tested in the similar context (No reminder). Finally, we expected that, regardless of test order, discrimination will be observed when pre-exposed to two similar contexts. That is, in the pre-exposure BC group we expected higher responding in the conditioning context compared to the generalization contexts both when first tested in the conditioned context (Reminder) and when first tested in the similar context (No reminder).

## Methods and Materials

### Participants

Participants (*n* = 34; 28 female, 6 male) were healthy undergraduate students, who ranged in age between 19 and 26 years (*M* = 22.41, *SD* = 1.88). For participation they received either partial course credit or a small amount of money (24 euros). All participants gave informed consent and were informed that they could withdraw from participation at any time. The experimenter briefly screened the participants to assure they were free from any contraindication for the study (see Experiment 1). The study was approved and in accordance with the guidelines of the Ethics Committee of the KULeuven. Participants were randomly assigned to the pre-exposure BC (*n* = 17) or the pre-exposure BX (*n* = 17) group. Due to technical difficulties startle data from two of these participants on day 2 (pre-exposure BX condition) and startle data from one subject on day 3 (pre-exposure BX condition) were excluded from analysis.

### Apparatus

#### Stimuli

**Figure 3:**
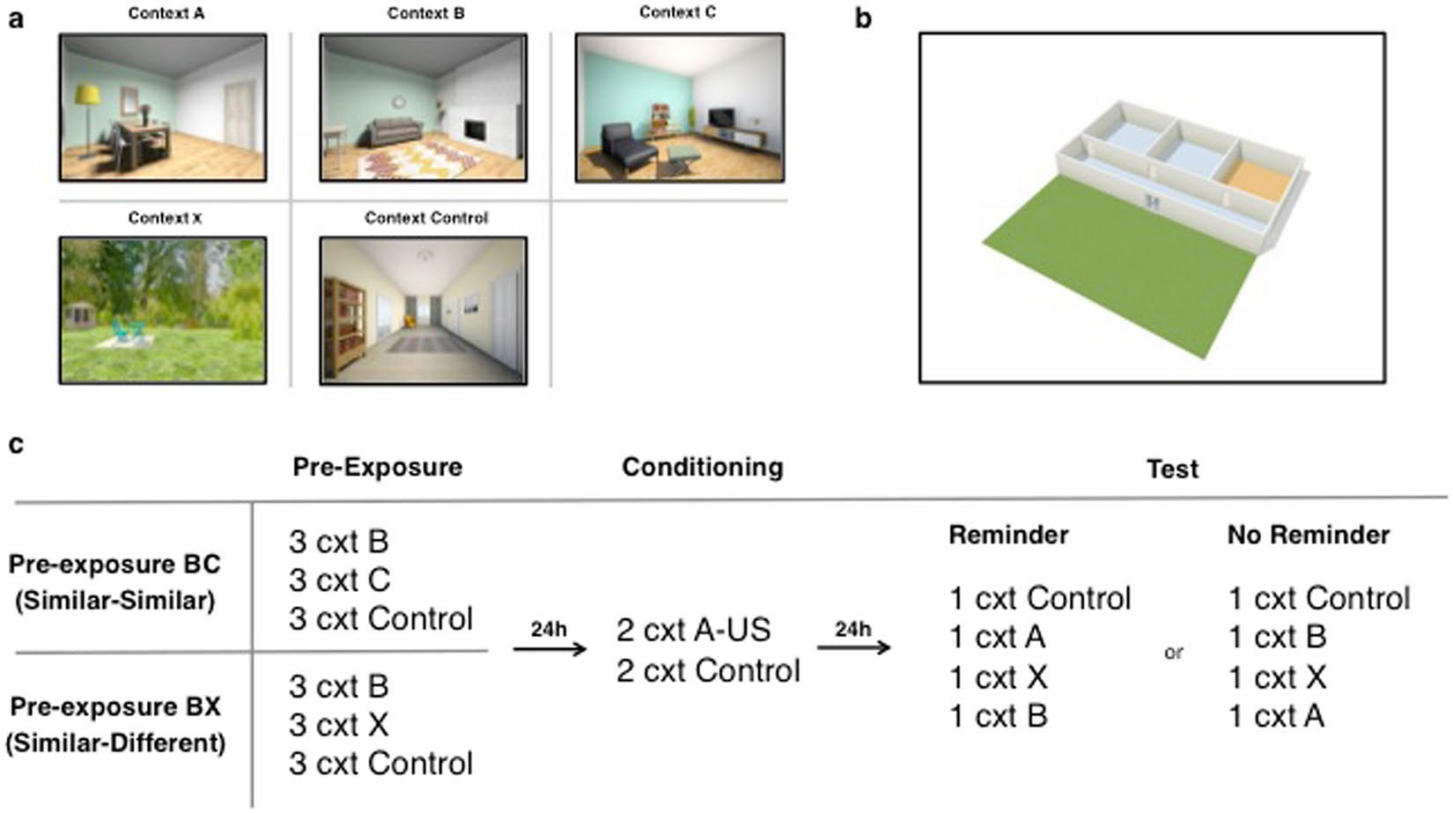
Overview of stimuli and experimental procedure for Experiment 2. Contextual stimuli (**a**), floor plan of the house (**b**), and schematic representation of the experimental design (**c**).

We used the same images as contexts A, B, and X and created an additional context C and a new image of the hallway (Fig. 3a,b). The same living room images served as context A and B, and the same image of the garden served as context X. A newly created living room image served as context C. The floor plan of the house was adjusted accordingly (www.floorplanner.com). Context A served as the threatening context; context B and context C as the similar contexts and context X as the context completely different from the threatening context. Again, the control stimulus consisted of an image of a hallway. In the previous experiment we observed enhanced startle responding to the control context during pre-exposure. The observation that darkness facilitates the startle response^24^ could explain this effect, given that the control stimulus was darker than the other stimuli. For the current experiment we designed a new control context image. Assignment of the living room images as context A, B, or C was counterbalanced across all participants. Stimulus presentation was controlled by Affect4 software designed at the University of Leuven (free download^25^).

#### Unconditioned Stimulus

The US was a 2ms electrocutaneous stimulus administered to the wrist of the left hand. Electrical stimulation was delivered through a pair of surface Sensormedics electrodes (10 mm diameter) filled with K-Y gel with inter-electrode mid-distance of 45 mm. Shock deliverance was controlled by a Digitimer DS7A constant current stimulator (Hertfordshire, UK)^26^.

#### Fear potentiated startle (FPS)

Startle response was measured through electromyography (EMG) of the right orbicularis oculi muscle. Two Ag/AgCl sensormedics electrodes (2.5 mm diameter) filled with TECA electrolyte gel were positioned approximately 1 cm under the pupil and 1 cm below the lateral canthus, respectively; a ground electrode was placed on the forehead, 1 cm below hairline^19^. The startle probe was a 50ms duration noise burst (100 dB) with instantaneous rise time presented binaurally through headphones. The raw signal was amplified by a Coulbourn isolated bioamplifier with a bandpass filter (v75-04) to reduce interference from other recorded signals (SCR). The recording bandwidth of the EMG signal was between 10 Hz and 20 kHz (plusminus 3 dB). Signal measuring for startle presentation was 1500 ms, starting 500 ms before probe onset. The EMG signal was digitized at 1000 Hz. The signal was rectified online and smoothed by Coulbourn multifunction integrator (v76-23 A) with a time constant of 50 ms. The data were band-pass filtered (13–500 Hz) online. The startle data were treated offline with PSPHA, a modular script based program for analysing psychophysiological data^27^. Peak amplitudes were defined as the maximum of the response curve within the 30–150 ms interval after the probe onset. Each peak amplitude was scored by subtracting its baseline score (=averaged EMG level between 1 and 20 ms after the noise probe onset).

#### Skin conductance level (SCL)

Electrodermal activity was measured with a skin conductance coupler manufactured by Coulbourn Instruments (model V71-23, Allentown, PA). Two 8 mm Ag/AgCl electrodes filled with K-Y gel were attached to the palm of the left hand. The coupler applied a constant voltage of 0.5 V across the electrodes. The resulting skin conductance signal passed through a Labmaster DMA 12 bit analog-to-digital converter (Scientific Solutions, Solon, Ohio) and digitized at 10 Hz from 2 s prior to context onset until 2 min after context onset. Skin conductance level was determined at 0.5 s intervals, both in the −2 to 0 s baseline window and the 0–10 s window after context onset. Scores for the entire-interval response (EIR) for this window were calculated by subtracting the mean SCL for the 2 s baseline immediately preceding context onset from the highest SCL value recorded during the 0–10 s window after context onset^28^. Note that in the 0–10 s window after context onset no startle probes and USs were delivered.

#### Online US-expectancy

Continuous online US-expectancy was measured throughout the experiment (for details see Experiment 1).

### Procedure

The procedure was the same as described for Experiment 1, except for pre-exposure on day 1 (Fig. 3c). Instead of pre-exposure to one context (A, B, or X) participants were now pre-exposed to two similar contexts (B and C) or to two contexts of which one was similar and the other different from the threatening context (B and X). Every context (B, C/X, control) was presented once per block (total of three blocks). The control context was presented first, followed by random presentation of the other two contexts. In Experiment 1 startle probes were delivered with a fixed interval. Previous studies used varying intervals for startle probe presentation to prevent fast habituation of the startle response^26,29^. Therefore, in the current experiment there were three different intervals for startle probe presentation^26^. Probes were delivered at 33, 50, 90 s or 10, 53, 115 s or 48, 60, 100 s after context onset. Assignment of startle presentation protocol during each context was random. On day 3 participants were assigned to one of two test orders: control, A, X, B or control, B, X, A. Thus with test order control, A, X, B participants received a reminder of the conditioned context before test in the generalization contexts. With test order control, B, X, A the participants were not reminded of the conditioned context but immediately tested in the generalization contexts. No shocks were delivered on the third day of testing. Note that additional context C was not tested on day 3. While we expected that pre-exposure effects on generalization to context C would not differ from effects to context B, we did not test this hypothesis. For sake of experimental rigor we included the same contexts on test as featured in Experiment 1.

### Data analysis

Participants were classified as non-responders when SCL on more than 75% of trials was below 0.1 (*n* = 4). This resulted in *n* = 16 participants in the pre-exposure BC group and *n* = 14 participants in the pre-exposure BX group for SCR analyses. SCR outliers (0.03%) were identified and removed based on within-participants *Z*-scores (*Z* > 2.5). SCR was mean corrected, to equalize the opportunity for each subject to contribute to the group mean^30^. The mean value used for correction was based on the all pre-exposure, conditioning and test trials. FPS data were T-transformed to control for inter-individual differences in startle responsiveness. Startle responses were averaged for each context presentation. US-expectancy ratings on day 2 and day 3 were averaged over the 2 min context presentation. Analyses of US-expectancy ratings, SCR and FPS were similar to Study 1, but now the additional between-subjects factor test order was added to the analyses.

SCR and FPS during pre-exposure on day 1 were tested with a mixed analysis of variance for repeated measures (ANOVA) with context (pre-exposure context; control context) and trial (trial 1; trial 2) as within-subjects factors; group (pre-exposure BC; pre-exposure BX) was included as between-subjects factors to test for differences between groups during pre-exposure. US-expectancy was not measured during pre-exposure. To test acquisition of US-expectancy, SCR, and FPS on day 2, data were subjected to a mixed ANOVA with context (context A; context control) and trial (trial 1; trial 2) as within-subjects factors; group (pre-exposure BC; pre-exposure BX) was included as between-subjects factors to test for differences in acquisition. To demonstrate that differential US-expectancy ratings between the conditioned context A and the control context persisted from acquisition (day 2) to test (day 3) a mixed ANOVA with context (context A; control context) and trial (last acquisition trial day 2; test trial day 3) as within-subjects factors and group (pre-exposure BC; pre-exposure BX) as between-subjects factors and was performed.

Further analyses were performed for US-expectancy, SCR, and FPS. To test whether generalization from the conditioned context A to the similar context B on day 3 differed between groups a mixed ANOVA with context (context A; context B) as within-subjects factor and group (pre-exposure BC; pre-exposure BX) as between-subjects factors was performed. Following up on a significant interaction with group paired-samples t-tests were performed to compare responding in context A with responding in the context B in the two groups separately. To test generalization from the conditioned context A to the different context X, the same analyses were performed with context X instead of context B.

Finally, post-hoc analyses to investigate the effect of test order on overgeneralization were conducted. To this end, test order was added as between subjects factor; a mixed ANOVA with context (context A; context B) as within-subjects factor and group (pre-exposure BC; pre-exposure BX) and test order (Reminder; No reminder) as between-subjects factors was performed. Following up on a significant interaction between group and test order, paired-samples t-tests comparing responding to context A and B were performed in the groups separately (pre-exposure BC-Reminder; pre-exposure BC-No reminder; pre-exposure BX-Reminder; pre-exposure BX-No reminder). The same analyses were performed to investigate generalization from the conditioned context A to the different context X, replacing context B with context X. The alpha level was set at 0.05 for statistical analyses. A Greenhouse-Geisser procedure was used in case of violation of the sphericity assumption in ANOVAs.

### Data availability

The programmed experiment (including stimulus materials) and the datasets analysed during the current study are available in the Open Science Framework (OSF) repository, https://osf.io/2dcp4/?view_only=05f27542f4144497893f2c94453b3857.

## Results

### Participants

There were no differences in US-intensity and US-evaluation (*F*_s_ < 1.05). There was a trend difference in reported trait anxiety (*F*_(1, 33)_ = 3.17, *p* = 0.085). Note that state anxiety did not differ between the groups(*F*_(1, 33)_ < 2.22) (Table 2). For analyses on differential responding (US-expectancy, SCR, and FPS) between the conditioned context and control context during conditioning (day 2) and test (day 3) see Supplementary materials. For analyses of SCR and FPS during pre-exposure see Supplementary materials.

**Table 2:** Mean values (SD) of the US-intensity, US-evaluation, state anxiety (STAI-S), and trait anxiety (STAI-T) for the pre-exposure BC (*n* = 17) and the pre-exposure BX (*n* = 17) conditions.

	Pre-exposure BC	Pre-exposure BX
US-intensity (mA)	15.5 (8.3)	12.4 (9.5)
US-evaluation	−2.5 (1.8)	−2.5 (0.9)
State anxiety	29.9 (6.3)	33.3 (7.0)
Trait anxiety	32.8 (8.6)	37.4 (6.2)

### US-expectancy

#### Generalization to the similar context (context B)

**Figure 4:**
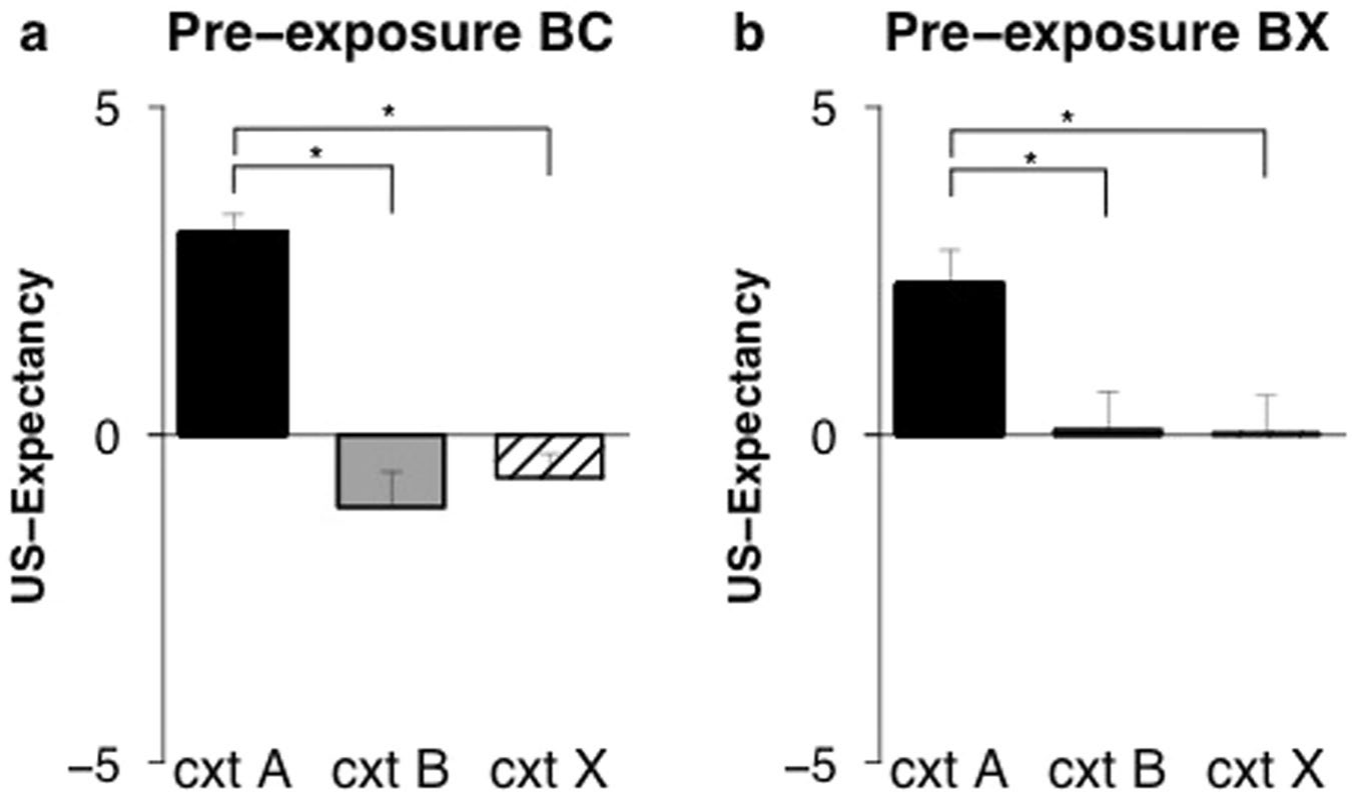
Pre-exposure to two similar contexts prevents enhanced generalization of US-expectancy. Mean US-expectancy ratings to context A, context B, and context X on test (day 3) for the pre-exposure BC (*n* = 17) (**a**) and the pre-exposure BX (*n* = 17) (**b**) conditions. Error bars represent s.e.m.

Differential ratings to the threatening context (context A) and the similar context B were greater in the pre-exposure BC group compared to the pre-exposure BX group (context x group; *F*_(1, 32)_ = 5.01, *p* < 0.032, *η*²_p_ = 0.14) (Fig. 4a,b). This indicates, in line with our expectations, that there was less generalization in those pre-exposed to two similar contexts (pre-exposure BC) relative to those pre-exposed to a similar and a different context (pre-exposure BX). Analyzing the groups separately showed that ratings to the conditioned context were higher than ratings to the similar context in the pre-exposure BC group (*t*_(16)_ = 11.04, *p* < 0.001, *d* > 2) (Fig. 4a). Hence, as expected there was good discrimination when pre-exposed to the similar contexts. Finally, contrary to expectation, there was also higher US-expectancy in the conditioned context compared to the similar context and in the pre-exposure BX group (*t*_(16)_ = 2.81, *p* = 0.013, *d* = 0.68) (Fig. 4b). Thus, while discrimination between contexts was more pronounced in the pre-exposure BC group, discrimination was also observed in the BX group.

#### Generalization to the different context (context X)

Discrimination between the conditioned context and the different context X was increased in the pre-exposure BC group compared to the pre-exposure BX group (context x group; *F*_(1, 32)_ = 4.71, *p* < 0.037, *η*²_p_ = 0.13) (Fig. 4a,b). Analyzing the groups separately showed that ratings to the conditioned context were higher than ratings to the different context in the pre-exposure BC group (*t*_(16)_ = 14.00, *p* < 0.001, *d* > 2) (Fig. 4a) and in the pre-exposure BX group (*t*_(16)_ = 3.66, *p* = 0.002, *d* = 0.89) (Fig. 4b). Thus, pre-exposure to two similar contexts resulted in more pronounced discrimination between contexts, while discrimination could also be observed when pre-exposed to two different contexts.

Similar to experiment 1, overgeneralization to the similar and the different context was still observed when participants were pre-exposed to a similar and a different context. However, this increase in generalization was prevented by pre-exposure to two similar contexts (B and C).

### Skin conductance response (SCR)

#### Test

**Figure 5:**
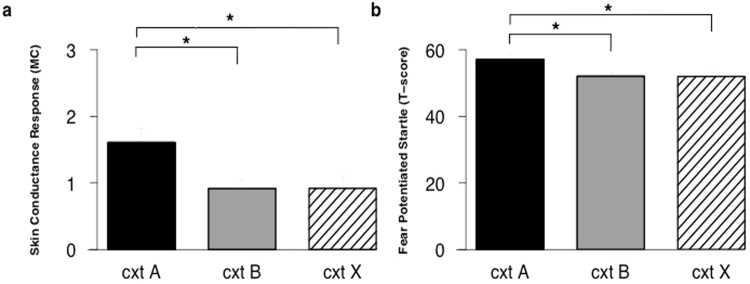
Pre-exposure does not affect skin conductance response (SCR) or fear potentiated startle (FPS). Mean skin conductance response (**a**) and mean startle response (**b**) to context A, context B, and context X on test (day 3). In absence of group differences, responses in the pre-exposure BC and pre-exposure BX groups are averaged (*n* = 33). Error bars represent s.e.m.

The groups did not differ in generalization of SCR from the conditioned context to the similar context B on test (context × group; *F*_(1, 26)_ < 1, *p* = 0.89, *η*²_p_ = 0.00). Overall, there was higher responding to the conditioned context compared to the generalization context B (*t*_(27)_ = 3.33, *p* = 0.003, *d* = 0.63) (Fig. 5a). There was a trend for a difference between groups in generalization of SCR from the conditioned context to the different context X on test (context x group; *F*_(1, 26)_ = 3.63, *p* < 0.07, *η*²_p_ = 0.12). Responding to the conditioned context was higher than responding to the different context X (*t*_(27)_ = 4.32, *p* < 0.001, *d* = 0.82) (Fig. 5a).

### Fear potentiated startle (FPS)

#### Test

The groups did not differ in generalization of FPS from the conditioned context to the similar context B on test (context x group; *F*_(1, 31)_ < 1, *p* = 0.76, *η*²_p_ = 0.00). Also, there was no difference between groups in generalization from the conditioned context to the different context X on test (context x group; *F*_(1, 31)_ < 1, *p* = 0.58, *η*²_p_ = 0.01). In general, startle responding to the conditioned context was higher than responding in the similar context B (*t*_(32)_ = 2.91, *p* = 0.006, *d* = 0.51) (Fig. 5b). Finally, responding in the conditioned context was higher than responding in the different context X on test (*t*_(32)_ = 3.27, *p* = 0.003, *d* = 0.57) (Fig. 5b).

### Exploratory analyses

Overgeneralization of US-expectancy could be prevented by pre-exposure to two similar contexts (pre-exposure BC group). However, participants who where pre-exposed to a similar and a different context still showed overgeneralization (pre-exposure BX group). Here, we will further investigate whether the increase in generalization, if not prevented, could be reduced by a reminder of the conditioned context. To this end, we will re-analyze the US-expectancy data with test order (Reminder vs. No reminder) as additional between-subjects factor. Note that SCR and FPS were not subjected to re-analysis. For these measures we did not observe any effect of pre-exposure. There was discrimination between the conditioned context and the generalization contexts on test irrespective of pre-exposure. Hence, in absence of overgeneralization, there is no need for an improvement in discrimination between the contexts.

### Test order effects on US-expectancy ratings

#### Generalization to the similar context (context B)

**Figure 6:**
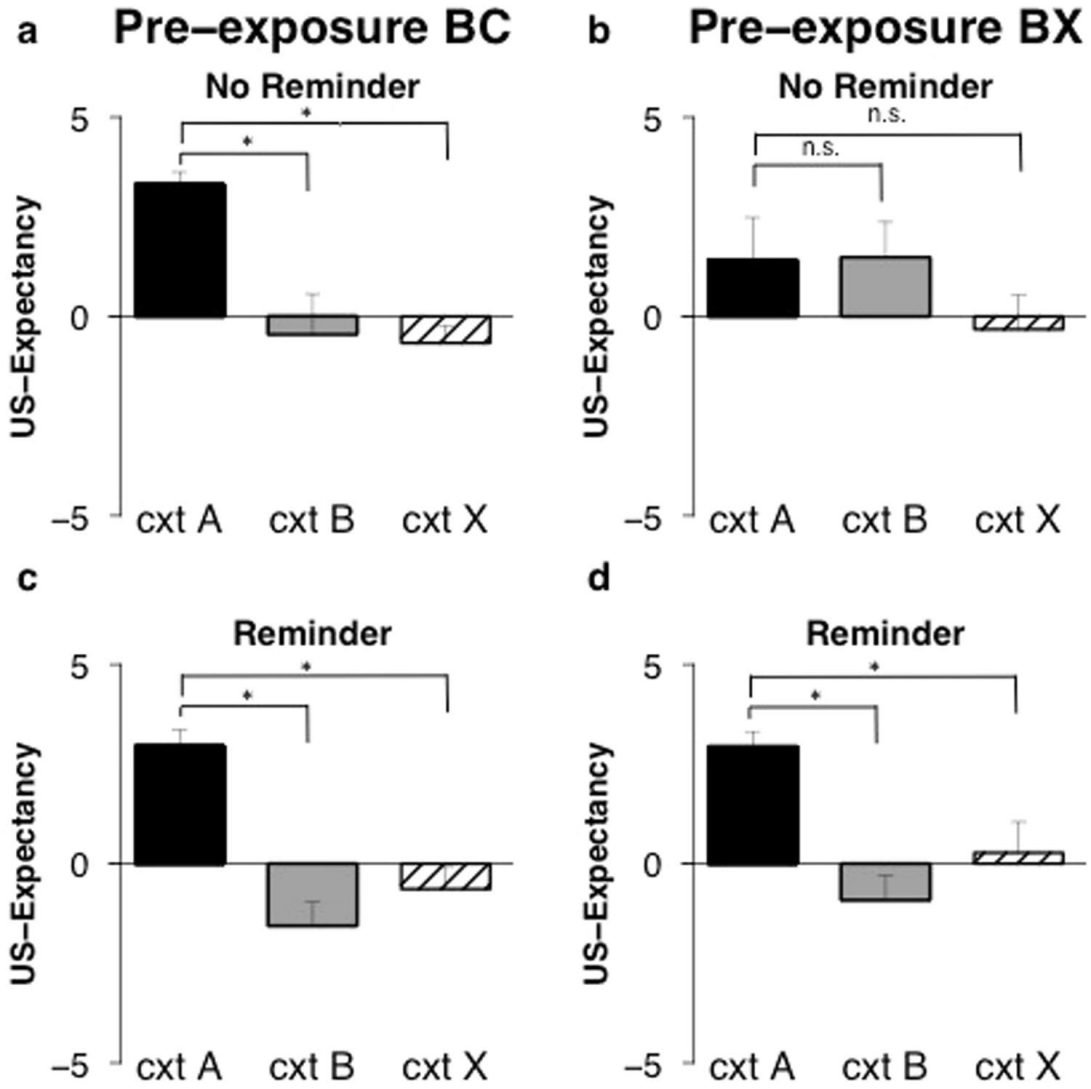
A reminder of the conditioning context reduces enhanced generalization. Mean US-expectancy ratings to context A, context B, and context X on test (day 3) for the pre-exposure BC (two similar contexts) and pre-exposure BX (similar and different contexts) groups separated for test order. For those first tested in context B (No reminder) this resulted in *n* = 7 in the pre-exposure BC (**a**) and *n* = 7 participants in the pre-exposure BX (**b**) conditions. For those first tested in context A (Reminder) this resulted in *n* = 10 in the pre-exposure BC (**c**) and *n* = 10 participants in the pre-exposure BX (**d**) groups. Error bars represent s.e.m.

Analysis revealed an interaction between context, group and test order (*F*_(1, 30)_ = 4.36, *p* < 0.045, *η*²_p_ = 0.13). Follow-up analyses were performed to compare differential ratings to the conditioned context and the similar context for the groups separately. In line with expectations, there was higher responding to the conditioned context compared to the similar context in the pre-exposure BC group (Fig. 6a,c), both when participants were reminded of the conditioned context (pre-exposure BC-reminder) (*t*_(9)_ = 11.11, *p* < 0.001, *d* > 2) (Fig. 6c) and when participants were not reminded (pre-exposure BC-no reminder) (*t*_(6)_ = 5.17, *p* = 0.002, *d* = 1.97) (Fig. 6a). Hence, pre-exposure to two similar contexts was already sufficient to reduce generalization to the similar context. In contrast, when pre-exposed to two different contexts, there was no discrimination between contexts when first tested in the similar generalization context (pre-exposure BX-no reminder) (*t*_(6)_ = −0.06, *p* = 0.96, *d* = 0.02) (Fig. 6b). Only in those participants that were reminded of the conditioned context did we observe discrimination between the contexts on test (pre-exposure BX-reminder) (*t*_(9)_ = 9.58, *p* < 0.001, *d* > 2) (Fig. 6d). Thus, overgeneralization as a result of pre-exposure was reversed by a reminder of the conditioned context.

### Generalization to the different context (context X)

There was no interaction with test order for generalization to the different context (context x test order; context × group × test order; *Fs*_(1, 30)_ < 1, *p*_*s*_ > 0.35, *η*²_p_ < 0.03). Note that when analysing the four groups separately, we observed a pattern of responding that is similar to generalization to the similar context (see section above). Ratings to the conditioned context A were higher than ratings to the different context X in those pre-exposed to the two similar contexts (pre-exposure BC group) (Fig. 6a,c) both when reminded of the conditioned context (pre-exposure BC-reminder) (*t*_(9)_ = 8.13, *p* < 0.001, *d* > 2) (Fig. 6c) and when participants were not reminded (pre-exposure BC-no reminder) (*t*_(6)_ = 22.27, *p* < 0.001, *d* > 2) (Fig. 6a). In contrast, when pre-exposed to different contexts, only the group that was reminded of the conditioned context showed discrimination between the conditioned context and the different context X (pre-exposure BX-reminder) (*t*_(9)_ = 4.76, *p* < 0.001, *d* = 1.51) (Fig. 6d). In the group that was not reminded, there was no discrimination between the contexts (pre-exposure BX-No reminder) (*t*_(6)_ = 1.31, *p* = 0.24, *d* = 0.49) (Fig. 6b). Hence, we still observed overgeneralization to the different context X when not reminded of the conditioned context. The increase in generalization was reduced by a reminder.

### Conclusion experiment 2

Overgeneralization of US-expectancy ratings to the similar context could be prevented and was not observed when pre-exposed to two similar contexts (pre-exposure BC group). Furthermore, overgeneralization of US-expectancy was still present in those pre-exposed to the similar and different context (pre-exposure BX group) but could be reduced by a reminder of the conditioned context. Again, we observed a pattern of responding for generalization to the different context that follows that of generalization to the similar context. Overgeneralization to the different context X was prevented by pre-exposure to two similar contexts. If it was not prevented (pre-exposure BX group), post-hoc analyses indicated that the increase in generalization could be reduced by a reminder of the conditioned context. There was no effect of pre-exposure on psychophysiological measures (SCR and FPS). Regardless of pre-exposure, there was good discrimination between the conditioned context and both the similar context (B) and the different context (X). Hence, generalization of SCR and FPS was neither enhanced, nor needed prevention/reduction.

## Discussion

The current study showed that generalization of US-expectancy could be induced and restrained in humans. Pre-exposure to a context that was similar to the conditioned context resulted in overgeneralization of US-expectancy ratings. In contrast, pre-exposing participants to two contexts that were similar to the conditioned context prevented increased generalization of US-expectancy. Increased generalization of contextual fear was no longer observed. Promisingly, once overgeneralization was induced by pre-exposure we were able to regain memory accuracy. A reminder of the conditioned context reduced increased generalization of US-expectancy ratings.

Notably, pre-exposure did not affect generalization of skin conductance response or fear potentiated startle. The dissociation in underlying neural mechanisms might explain these differences. Startle responding is mediated by the amygdala^31,32^, while declarative memory (US-expectancy) is thought to be largely dependent on the hippocampus^33,34^, the structure that is also known to be responsible for pattern completion (the ability to recall memories from partial cues)^35,36^. A memory representation adapts more easily to small changes in the environment when it relies on the hippocampal circuitry and pattern completion processes. When behaviour depends on extra-hippocampal areas (i.e., fear potentiated startle), it is more conservative about change. This might explain the dissociation in results between hippocampal-dependent US-expectancy ratings and the amygdala-mediated startle response. Note that the underlying mechanisms of SCR remain relatively unknown. In contrast to the current findings, it is often observed that SCR runs parallel to the contingencies^37^.

A previous animal study showed that pre-exposure techniques could be used to prevent generalization^9^. Generalization of contextual fear typically increases over time, due to a decay of contextual details (recently this was also observed in humans^23^). The representation of the conditioned context degrades and it becomes more difficult to discriminate between the conditioned context and a similar context. To prevent the increase in generalized fear a stronger representation of the conditioned context was induced by repeatedly pre-exposing the animals to this context. Indeed, this pre-exposure training prevented the time-induced enhanced generalization to the similar context^9^. While the current study also demonstrated that pre-exposure could be used to prevent overgeneralization, there is an important difference between the studies. In the study by Biedenkapp and Rudy (2007) pre-exposure established a stronger representation of the conditioned context, which increased the ability to discriminate between that context and a similar context at test. The prevention procedure in the current study was designed in the first place to increase the ability to differentiate between similar contexts during conditioning - and not test -, such that the similar representation was not recalled. As a consequence, this resulted in differentiation between the contexts on test. In contrast, the treatment strategy explored in Experiment 2 specifically aimed to increase differentiation on test. Prevention of recall of the pre-exposure context during conditioning was no longer possible, but a reminder of the conditioned context increased discrimination between contexts.

In a different line of research, pre-exposure has been employed to affect conditioning^38,39^. Animal studies showed that pre-exposure to the threatening context retards the development of conditioning, a phenomenon known as latent inhibition. To our knowledge there have been only two studies conducted on latent inhibition of contextual conditioning in humans. One study did not find an effect of context pre-exposure^22^, while the other study revealed only a very preliminary latent inhibition effect^23^. In the current study there was no evidence of latent inhibition given that conditioning or retention of conditioning (from day 2 to day 3) did not differ when participants were pre-exposed to the conditioned context (pre-exposure A condition) compared to the participants in the other groups (Experiment 1). It has been suggested that only long lasting pre-exposure sessions attenuate context conditioning^40^, explaining the lack of an effect in the current study. Note that this explanation is hard to reconcile with the study by Biedenkapp and Rudy (2007) that showed that repeated pre-exposure to the to-be conditioned context prevented overgeneralization over time but did affect conditioning itself.

In the current study increased generalization was inferred from an absence of differential US-expectancies between the conditioned context and generalization context B on test. Visual inspection suggests that this overgeneralization does not necessarily go together with an overall increase in US-expectancy. These findings are in line with a series of studies on cued fear conditioning and overgeneralization^3,41–43^. In these studies it was demonstrated that patients suffering from different anxiety pathologies showed flattened generalization gradients relative to healthy controls. While it was not tested, a pattern of reduced responding to the threatening stimulus and increased responding to the safety stimulus can be observed across studies. Thus, similar to the current study, overgeneralization is characterized by reduced discrimination rather than an overall increase in responding. Furthermore, due to overgeneralization more stimuli become predictive of the aversive consequence (i.e., not only the conditioned context but also the similar context). In absence of a single predictor, it is plausible that confidence of the contingencies is reduced. Indeed, participants indicated to be less sure about the conditioned context as a predictor of the US at test.

The reduction in responding to the conditioned context may also explain the unexpected finding of overgeneralization to the different context. A previous animal study showed that recall of pre-exposure context during conditioning results in a specific link of the recalled representation and shock that does not extend to a completely different context^4^. Alternatively, our results could suggest that rather than establishing a specific link between the retrieved pre-exposure context and shock^4–6^, recall of the pre-exposure context during conditioning resulted in a more generic memory representation of the conditioned context. Thus, instead of two separate representations that are associated with shock, the two representations blended into a single representation. Then, loss of specific features of the threatening context might have facilitated generalization to less similar contexts.

From a clinical point of view, the current study demonstrates that anxiety is not restricted to stimuli present during trauma. Here, we show that overgeneralization, a robust marker of anxiety pathology^1–3^, could already be induced in healthy participants by means of their previous experiences. Promisingly, we were able to prevent increased generalization by pre-exposure to multiple contexts that were similar to the conditioned context. This is an encouraging first step towards the development of effective techniques to prevent fear generalization. Such interventions might be used in those groups in which traumatic experiences are likely to occur. Otherwise, after a fear-learning event interventions aimed at reducing generalization should be applied.

In conclusion, this study showed that pre-exposure could result in contextual overgeneralization of US-expectancy. But we offered two different strategies to curb overgeneralization. First, pre-exposure to a pair of similar contexts prevented increased generalization. Second, if not prevented, memory accuracy for contexts could be improved by a return to the conditioned context, resulting in reduced generalization of US-expectancy. These findings advance our insight in memory dynamics and lay a foundation for the development of interventions specifically aimed at generalization reduction.

## Electronic supplementary material

Supplementary Material
