# Supplementary material for: Prevention and treatment strategies for contextual overgeneralization
Source: Sci Rep. 2017 Dec 5;7:16967. doi: 10.1038/s41598-017-16893-2 (PMC5716998; doi:10.1038/s41598-017-16893-2)
Supplement: Supplementary file 1 — Supplementary Material [file 41598_2017_16893_MOESM1_ESM.pdf]

Supplementary Information for

Prevention and treatment strategies for contextual overgeneralization

Dieuwke Sevenster, Kim Haesen, Bram Vervliet, Merel Kindt, & Rudi D'Hooge

Experiment 1. Pre-exposure induces contextual overgeneralization in humans

Results

US-expectancy ratings. Analysis revealed an increase in differential US-expectancy ratings from the first to the second trial of conditioning (context x trial; $F_{(1, 38)}$ = 117.79, p < .001, η^2_p = .76). There was no difference between groups in context conditioning (context x trial x group; $F_{(1, 38)}$ < 1, p = .62, η^2_p = .03) (Supplementary Fig. S1A-C).

Test. There was a trend for an increase in differential responding between the conditioned context and the control context from the last trial of acquisition (day 2) to the test trial (day 3) (context x trial; $F_{(1, 38)}$ = 3.04, p = .089, η^2_p = .07), indicating that acquired differential ratings persisted during test. The groups did not differ in differential ratings from acquisition to test (context x trial x group; $F_{(2, 38)}$ < 1, p = .39, η^2_p = .05).

Fear potentiated startle (FPS)

Pre-exposure. Contrary to expectation we observed higher startle responding to the control context compared to the pre-exposure context (main effect context; $F_{(1, 38)}$ = 25.23, p < .001, η^2_p = .40). Note that this difference was observed regardless of type of pre-exposure context (context A, B, or X), since there were no differences between groups in startle responding during pre-exposure (context x group; context x trial x group; F_s < 1.29, p_s >.29, η^2_p <.04) (Supplementary Fig. S2A-C).

Conditioning. Differential responding changed from the first to the second trial of conditioning (context x trial; $F_{(1, 36)}$ = 4.89, p < .033, η^2_p = .12) that did not differ between groups (context x trial x group; $F_{(2, 36)}$= 1.20, p > .31, η^2_p = .06). Startle responding to the control context was higher compared to the conditioned context on the first trial (main effect context; $F_{(1, 36)}$ = 7.08, p < .012, η^2_p = .16) and was absent on the second trial (main effect context; $F_{(1, 36)}$ < 1, p > .43, η^2_p = .02). Thus, the unexpected difference in startle responding during pre-exposure persisted on the first trial of conditioning and acquisition of startle responding was not observed (Supplementary Fig. S2A-C).

Test. The groups did not differ in generalization of startle responding from the conditioned context A to the similar context B (context x group; $F_{(2, 38)} < 1$, $p > .72$, $\eta^2_p = .02$) (Supplementary Fig. S3A-C). Also, there were no differences in generalization from the conditioned context A to the different context X (context x group; $F_{(2, 38)} < 1.27$, $p > .29$, $\eta^2_p = .06$). Regardless of group, responding to the conditioned context did not differ from responding in the similar context B ($t_{(40)} = -.34$, $p = .73$, $d = .05$) or the different context X on test ($t_{(40)} = -.74$, $p = .47$, $d = .11$) (Supplementary Fig. S3A-C).

Experiment 2. Preventing and reducing fear generalization in humans

Results

US-expectancy ratings

Conditioning. We observed context conditioning of US-expectancy ratings, evidenced by an increase in differential US-expectancy ratings from the first to the second trial of conditioning (context x trial; $F_{(1, 32)} = 102.30$, $p < .001$, $\eta^2_p = .76$). The groups did not differ in acquisition of US-expectancy (context x trial x group; $F_{(1, 32)} < 1$, $p = .23$, $\eta^2_p = .05$)(Supplementary Fig. S4A,B).

Test. There was an increase in differential responding between the conditioned context A and the control context from end of acquisition (trial 2, day 2) to the test trial (day 3) (context x trial; $F_{(1, 32)} = 9.01$, $p = .005$, $\eta^2_p = .22$), that did not differ between groups (context x trial x group; $F_{(1, 32)} < 1$, $p = .72$, $\eta^2_p = .00$). This indicates that acquired differential ratings persisted during test.

Skin conductance response (SCR)

Pre-exposure. Responding to the pre-exposure contexts and the control context did not differ between groups (context x group; context x trial x group; $F_s < 1.22$, $p_s > .31$, $\eta^2_p < .05$) (Supplementary Fig. S5A,B). We observed no difference in skin conductance responses to the pre-exposure contexts (B and C/X) and the control context on trials 1 to 3 of pre-exposure (main effect context; $F_{(2, 44)} < 1$, $p = .68$, $\eta^2_p = .02$) (Supplementary Fig. S5A,B).

Conditioning. Analysis revealed conditioning of SCR, evidenced by an increase in differential responding from the first to the second trial of conditioning (context x trial; $F_{(1, 23)}$ = 16.35, p < .001, η^2_p = .42). The groups did not differ in acquisition of SCR (context x trial x group; $F_{(1, 23)}$ < 1, p = .59, η^2_p = .01) (Supplementary Fig. S5A,B).

Fear potentiated startle (FPS)

Pre-exposure. Startle responding did not differ between the pre-exposure contexts (B and C/X) and the control context on trials 1 to 3 (main effect context; $F_{(2, 64)}$ < 1, p = .58, η^2_p = .02; context x group; $F_{(2, 64)}$ < 1, p = .74, η^2_p = .01)(Supplementary Fig. S6A,B).

Conditioning. There was a near-significant acquisition of FPS, evidenced by a change in differential responding from the first to the second trial of conditioning (context x trial; $F_{(1, 30)}$ = 3.98, p < .055, η^2_p = .12; context x trial x group; $F_{(1, 30)}$ < 1, p < .71, η^2_p = .01)(Supplementary Fig. S6A,B).

Supplementary Figures

<u>Experiment 1</u>

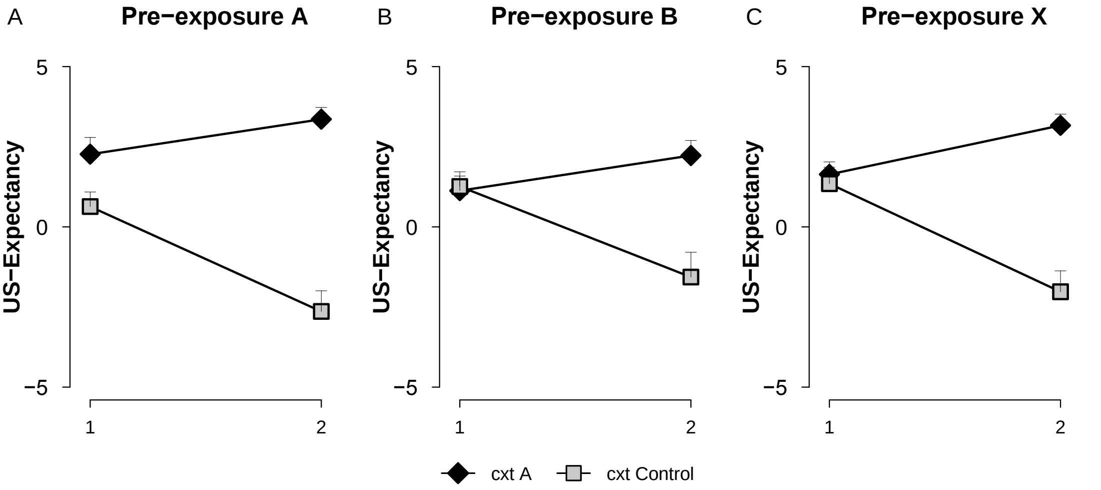

Figure S1. Mean US-expectancy ratings to context A and the control context during conditioning (day 2) for the pre-exposure A (n = 14) (**A**), pre-exposure B (n = 14) (**B**), and pre-exposure X (n = 13) (**C**) groups. Error bars represent s.e.m.

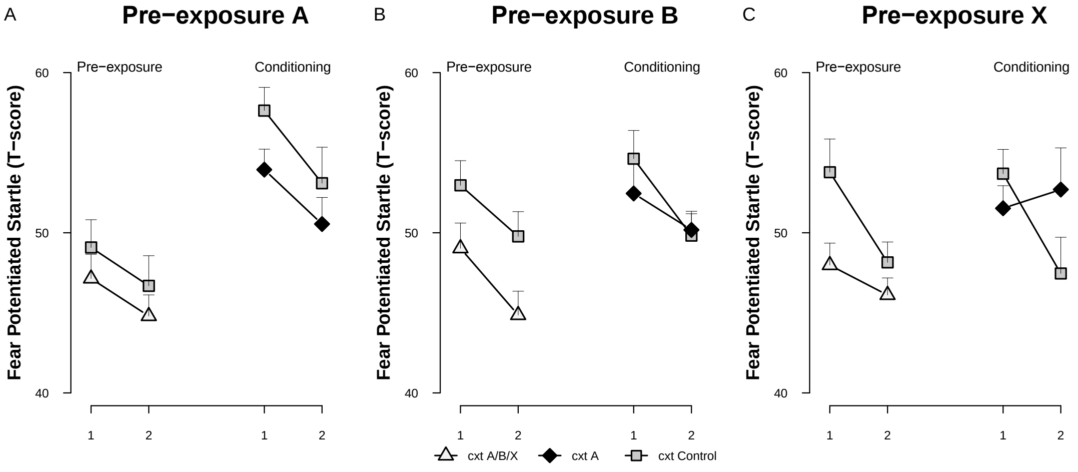

Figure S2. Mean startle response during pre-exposure on day 1 (pre-exposure contexts and control context) and startle responses to context A and the control context during conditioning on day 2 for the pre-exposure A (*n* = 14) (**A**), pre-exposure B (*n* = 14) (**B**), and pre-exposure X (*n* = 11-13) (**C**) groups. Light grey triangles represent responses to the pre-exposure context that differed between the groups (context A, context B, or context X); grey squares refer to the control context; black diamonds represent the conditioned context A. Error bars represent s.e.m.

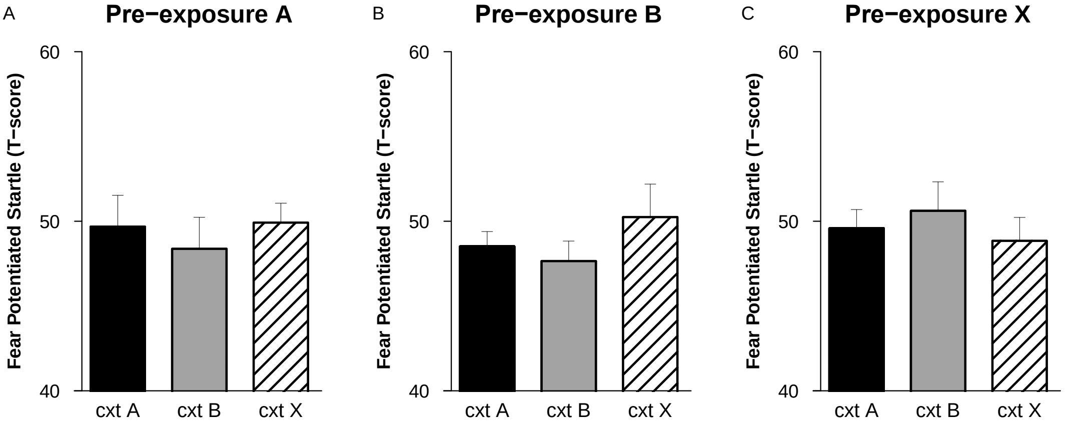

Figure S3. Mean fear potentiated startle (FPS) response to context A, context B, and context X on test (day 3) for the pre-exposure A (*n* = 14) (**A**), pre-exposure B (*n* = 14) (**B**), and pre-exposure X (*n* = 13) (**C**) groups. Error bars represent s.e.m.

Experiment 2

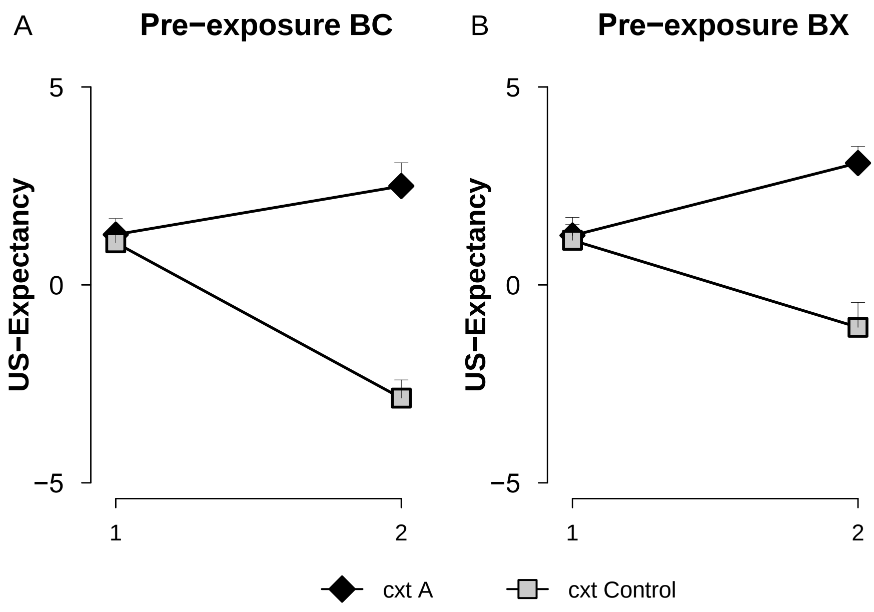

Figure S4. Mean US-expectancy ratings to context A and the control context during conditioning (day 2) for the pre-exposure BC (*n* = 17) (**A**) and pre-exposure BX (*n* = 17) (**B**) groups. Error bars represent s.e.m.

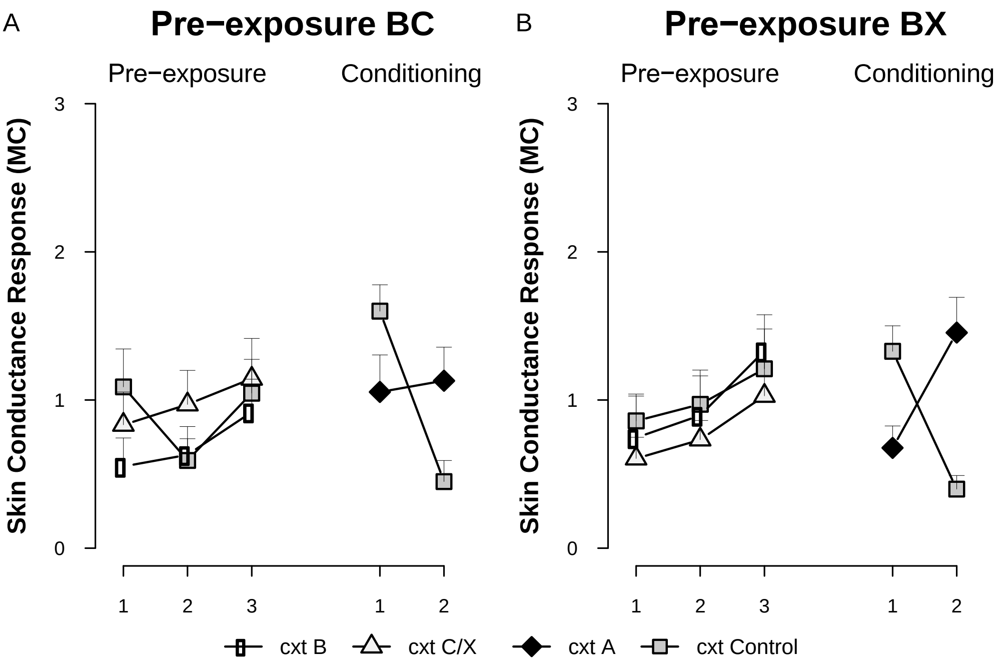

Figure S5. Mean skin conductance response (SCR) during pre-exposure on day 1 (pre-exposure contexts and control context) and responses to context A and the control context during conditioning on day 2 for the pre-exposure BC (*n* = 11-15) (**A**) and pre-exposure BC (*n* = 10-13) (**B**) groups. Light grey triangles symbolize responses to the pre-exposure context that differed between the groups (context C or context X), white circles represent context B that was presented in both groups; grey squares refer to the control context; black diamonds represent the conditioned context A. Error bars represent s.e.m.

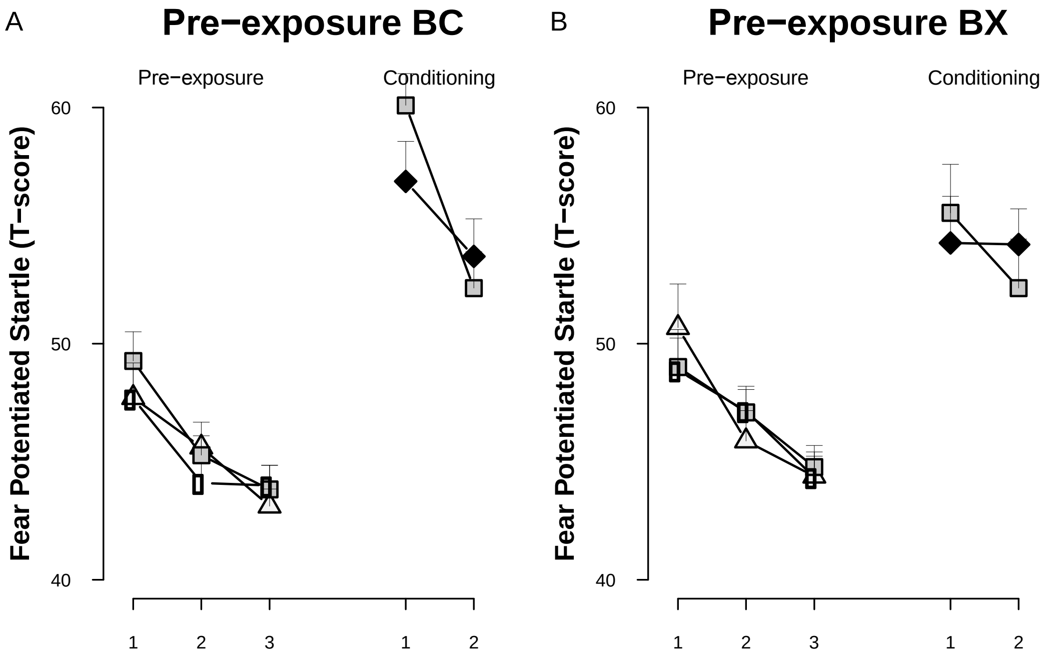

Figure S6. Mean startle response during pre-exposure on day 1 (pre-exposure contexts and control context) and startle responses to context A and the control context during conditioning on day 2 for the pre-exposure BC (*n* = 17) (**A**) and pre-exposure BX (*n* = 15-17) (**B**) groups. Light grey triangles represent responses to the pre-exposure context that differed between the groups (context C or context X); white circles symbolize responses to context B that was presented in both groups; grey squares refer to the control context; black diamonds represent the conditioned context A. Error bars represent s.e.m.
